# Carbon offsets, reversal risk and US climate policy

**DOI:** 10.1186/1750-0680-4-3

**Published:** 2009-06-15

**Authors:** Bryan K Mignone, Matthew D Hurteau, Yihsu Chen, Brent Sohngen

**Affiliations:** 1 The Brookings Institution, 1775 Massachusetts Avenue, NW, Washington, DC 20036, USA; 2 Center for Applied Macroeconomic Analysis, Australian National University, Canberra ACT 0200, Australia; 3 Western Regional Center of the National Institute for Climatic Change Research, Northern Arizona University, Box 6077, Flagstaff, AZ 86011, USA; 4 School of Engineering, University of California, Merced, 5200 North Lake Road, Merced, CA 95343, USA; 5 Agricultural, Environmental and Development Economics, The Ohio State University, 2120 Fyffe Road, Columbus, OH 43210-1067, USA; 6 Resources for the Future, 1616 P Street NW, Washington, DC 20036, USA

## Abstract

**Background:**

One controversial issue in the larger cap-and-trade debate is the proper use and certification of carbon offsets related to changes in land management. Advocates of an expanded offset supply claim that inclusion of such activities would expand the scope of the program and lower overall compliance costs, while opponents claim that it would weaken the environmental integrity of the program by crediting activities that yield either nonexistent or merely temporary carbon sequestration benefits. Our study starts from the premise that offsets are neither perfect mitigation instruments nor useless "hot air."

**Results:**

We show that offsets provide a useful cost containment function, even when there is some threat of reversal, by injecting additional "when-flexibility" into the system. This allows market participants to shift their reduction requirements to periods of lower cost, thereby facilitating attainment of the least-cost time path without jeopardizing the cumulative environmental integrity of the system. By accounting for market conditions in conjunction with reversal risk, we develop a simple offset valuation methodology, taking into account the two most important factors that typically lead offsets to be overvalued or undervalued.

**Conclusion:**

The result of this paper is a quantitative "model rule" that could be included in future legislation or used as a basis for active management by a future "carbon fed" or other regulatory authority with jurisdiction over the US carbon market to actively manage allowance prices.

## Background

The efficiency of the natural carbon sink is in decline, with land-use change contributing approximately 16% of annual carbon emissions from 2000–2006 [1]. While the future trajectory of land-use emissions will depend on a variety of uncertain factors, ranging from future patterns of rural development to the impacts of climate change on disturbance and forest health [2-5], the land-use contribution to total emissions will almost certainly remain quantitatively significant [2]. Consequently, architects of US cap-and-trade policies have sought to include instruments to reduce such emissions by issuing carbon offsets for projects that avoid expected emissions or deliberately sequester additional carbon [6]. Offsets are economically and politically attractive because, by expanding the scope of the program to include lower cost options, they increase the number of compliance opportunities in the market, exert downward pressure on carbon prices and minimize the overall social cost of abatement [7].

However, the wisdom of including large quantities of mitigation from outside the energy sector (and from the land-use sector, in particular) has been widely questioned by those concerned with a wide range of potential problems. These include potential future losses of carbon on-site that could result from natural disturbance, poor management or other factors (collectively referred to as "non-permanence") and potential losses of carbon off-site due to activity shifting or market price adjustments that drive up carbon losses elsewhere (typically called "leakage"). Existing regulations governing offset project development have attempted to account for the possibilities of non-permanence and leakage by applying a discount factor to permits commensurate with their perceived reversal risk. For example, the northeast Regional Greenhouse Gas Initiative (RGGI) discounts offset credits at 10% [8] and early versions of the Waxman-Markey bill discounted offset credits at 20% [6]. The Voluntary Carbon Standard requires that a proportion of the offset credits be deposited in a buffer pool, with that proportion determined by a categorical risk assessment [9].

While this approach internalizes some of the risks associated with such carbon assets, the discount factors used in practice are largely qualitative and fail to account for the economic value of such instruments. As a result, they raise the costs of forest carbon without acknowledging any of the benefits that risky carbon offsets might provide by mitigating upside price shocks and reducing overall mitigation costs. This paper shows how to quantitatively adjust the discount factor to account for these benefits. The resulting "model rule" could be used by regulators to dynamically adjust the supply of offsets in an emissions permit market to more finely manage carbon prices.

## Results

Typically, broad concerns over environmental integrity take two distinct forms, reflected in requirements that offsets be both "additional" and "permanent" [10]. The first criterion effectively requires that the activity under consideration yield emissions reductions that would not otherwise occur. Assignment of "additionality" therefore hinges on confidence in the relevant emissions baseline path, since this is the trajectory against which any reductions will be measured and against which credits will be awarded.

The second criterion requires that a project safely sequester carbon over the long time horizons demanded by the climate system itself. An offset is permanent, in an operational sense, if the emissions reduction does not reappear as a source in a later compliance period, although the proper length of this horizon is debatable. Offset credits from land-use projects are particularly important to evaluate in this context, because carbon stored in forests or other terrestrial systems could escape back into the atmosphere for a variety of reasons, ranging from natural disturbances (e.g., fire) to inadequate protection from human interference (e.g., logging) [11]. As discussed above, future losses of carbon off-site due to economic leakage could also jeopardize the effective permanency of stored carbon.

Reversals of either kind (on-site or off-site) are not problematic unless they fail to be properly internalized by the underlying crediting framework. In that case, permits may be awarded in excess of the net integrated emissions reductions specified by the policy. In a world in which the trajectory of carbon loss could be reasonably well anticipated, this outcome could be avoided in one of two ways. First, project developers or offset buyers could individually retain liability for future carbon loss, in which case those entities would be responsible for purchasing additional permits *ex post*, whenever sequestered carbon was shown (through monitoring) to have escaped.

A number of mechanisms to place liability on the buyer of offsets have been proposed. One is to create "rental contracts" for temporary sequestration [12]. Another is to issue so-called temporary sequestration credits (tCERs) under which holders of such credits must make up the carbon content of these credits in other ways after the term of the temporary credit expires [13]. Finally, credits for risky assets might be issued in the usual manner, but with the regulator imposing an additional condition that those holding such assets acquire private insurance just as vehicle owners are required to demonstrate proof of insurance before registration. Of course, this requirement is feasible only if there is an existing insurance market for offsets. For an example of how this might be implemented, see the model rule developed as part of the northeast Regional Greenhouse Gas Initiative [8].

Alternatively, the regulator could discount credits *ex ante *(at the time of certification) to account for anticipated future carbon loss, thus effectively transferring liability to itself. In this case, the government would issue less than one tradable permit for every ton of carbon sequestered (assuming each permit represents the legal right to emit one ton), thereby acquiring a greater number of permits up front to sufficiently compensate for future carbon losses. A similar objective could also be pursued through the creation of a "buffer pool" [9] rather than through direct discounting. While allowing private entities the opportunity to internalize risk is arguably more efficient than these approaches, some form of regulator discounting or "buffering" is likely to be needed in the short run before a robust offset insurance market develops and because private liability would be difficult to implement in the case of future off-site reversals.

While the failure to properly account for reversal risk leads to the possibility of *over*valuation, a failure to acknowledge the economic efficiency benefits of (even risky) offsets can lead to systematic *under*valuation. As an example, consider an offset project in which all of the carbon initially sequestered is lost in a future compliance period. Fully discounting carbon reversals, as above, would imply that no credits should be issued for such a project. However, if credits were initially issued at a time when permit prices in the market were high, and credits for future carbon loss were later surrendered at a time when prices were low, then this combined transaction would lower total abatement costs by allowing market actors to endogenously shift abatement across time in pursuit of an economically efficient outcome.

When liability for future carbon losses is privately held, those trading offsets must decide for themselves when these types of transactions offer credible arbitrage opportunities, given their own expectations about future prices. Under this type of regulation, firms would use a collection of compliance tools, consisting of both risky and non-risky assets, to navigate an efficient abatement path. In the alternative case in which credits are discounted up front, the regulator would need to determine an appropriate discount rate that incorporates both reversal risk *and *the benefits of increased when-flexibility. The regulator, however, would like to employ a discount rule that mimics, as much as possible, the incentives that individual actors would face were they themselves held liable for future carbon losses.

For example, suppose a project developer sequesters two tons of carbon in the present period when prices exceed the expected discounted long-run average price by a factor of two, knowing that all of the carbon stored initially will ultimately be lost within the relevant time horizon. In response to this submission, the regulating agency prints two permits, issues one to the project developer (effectively discounting at 50% from the developer's perspective) and retains one for itself. The regulator immediately liquidates its own permit in the secondary market and uses the revenue to buy back and retire two permits in the future, once the price drops back to (or below) the long-run average value. Because the regulator removes two permits from circulation in a future period, the carbon loss is properly internalized and the cumulative integrity of the program is not violated.

In the above example, the regulator is essentially constraining liquidity in the market when it believes that prices have risen to levels above those justified by fundamentals. This mechanism would thus complement firm-level borrowing decisions (assuming such actions were allowed by the policy) and help to collapse speculative bubbles early. Of course, any mechanism designed to enhance when-flexibility, whether realized through firm-level borrowing decisions or through the actions of a central regulator, necessarily requires judgments to be made about future market conditions, and prices in particular. A limited discretionary mechanism, like the one proposed here, simply provides an additional check on the judgments made by firms and spreads the decision about whether to shift abatement across a more diverse set of actors in the system.

Tightening and loosening constraints on offsets in real time and in response to actual market conditions has been proposed previously in the context of a "carbon fed" [14], but markets may behave better in this case if clear, quantitative rules are written up front. When it comes to risky carbon assets, the regulator's fundamental objective is to make sure that the crediting system correctly balances the tendency to overvalue offsets (by not sufficiently accounting for reversal risk and the implied risk to the climate system) with the tendency to undervalue them (by not sufficiently accounting for the benefit of when-flexibility). Within the broad class of risky assets, we contend that the underlying economic and environmental goals would be best served by explicitly quantifying reversal risk along a continuum and transparently and dynamically adjusting valuations on a project-specific basis to reflect the nature of such risk in the context of the broader carbon market. The application of such a valuation rule by a central authority would increase when-flexibility (lower compliance costs) without violating the cumulative environmental integrity of the system.

Consider the expected price path, P_e_(t) of carbon permits, derived, for example, from model estimates of a prescribed emissions reduction path [15]. In effect, this price path represents the "target price" that the regulator hopes to defend through the constrained use of offsets. Suppose offsets are allowed into the system in some period *t *= τ, but that a fraction *f *of the carbon initially sequestered is lost over the relevant time horizon. The parameter *f *in this model will inevitably vary by project or project type, but existing research provides ample guidance [16]. The immediate economic *benefit *(per ton of carbon) of allowing such credits into the market is the spot price, P_s_(τ), because that reflects the marginal (per ton) cost of an avoided permit. The total economic *cost *of using the offset is the sum of the deployment cost of the offset itself, C(τ), *and *the present value of the future permit that must be purchased at the future target price to compensate for the eventual carbon loss. Assuming that P_e_(t) rises at the long-term interest rate, the net present value of the cost is C(τ) + *f*·P_e_(τ).

It is worth noting that the full present value of the carbon loss term (assuming the fraction *f *is lost in time T) can be written as *f*·*P*_*e*_(τ)·*e*^*rT*^·*e*^-*rT*^. The exponentially increasing term represents the steadily increasing forward price on carbon (as forecast by most model assessments), and the declining exponential term represents the discount factor (with the discount rate also assumed to be equal to the long-term interest rate). Because the exponential terms cancel, the longer expression simplifies to *f*·*P*_*e*_(τ), which is what appears in the preceding paragraph.

The value of the offset V(τ) can be measured by one's willingness to pay for such an instrument, which in turn, can be found by solving for C(τ) when the total cost of using the instrument exactly balances the benefit of using it. This returns the maximum price at which offsets would be an attractive compliance vehicle: V(τ) = P_s_(τ) - *f*·P_e_(τ). Further dividing through by the spot price, the value of the offset *relative to the spot price *is δ = V(τ)/P_s_(τ) = 1 - *f*·P_e_(τ)/P_s_(τ). Since, from a cost containment perspective, we are most concerned with the case where P_s_(τ) > P_e_(τ), it is useful to define *S *as the relative price shock at t = τ, or equivalently, as the factor by which the spot price exceeds the target price, so that *S *= P_s_(τ)/P_e_(τ). This implies that δ = 1 - *f*/*S*. If δ is the relative value of the offset, it is also the factor by which a given ton of emissions should be discounted at the time of crediting.

## Discussion

**Figure 1:**
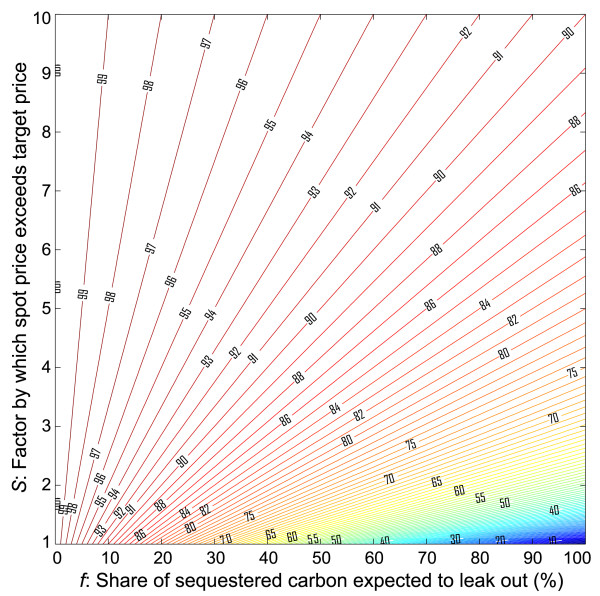
**Applied discount factor (δ) in percent as a function of the share of sequestered carbon expected to be lost over the relevant time horizon (*f*) and the factor by which the spot price for permits exceeds the target price set by the regulator (*S*) or by which it exceeds the expected discounted future equilibrium price**. The limits described in the main text are easy to identify on this figure. The no-reversal limit is found along the left edge of the figure; the discount factor of 100% is independent of the value of *S*. The full reversal limit returns a low discount factor when *S *is small (lower right-hand corner) but a high discount factor when *S *is large (upper right-hand corner).

The discount factor δ is plotted as a function of *f *and *S *in Figure 1. A few limiting cases are worth discussing explicitly. First, consider the case *f *→ 0, in which there is no reversal over the relevant time horizon. In that case, δ → 1, meaning that the offset credit should not be devalued at all at the time of crediting, regardless of market prices. That is, the credit should be treated like any other ton of emissions abatement from the energy sector.

For many activities related to changes in land management, 0 <*f *< 1, but the exact risk-adjusted value of *f *may not be known precisely. For simplicity, consider the alternative limit *f *→ 1, which implies that all carbon originally sequestered is ultimately lost. Arguably, this is a good approximation when the relevant time horizon is very large. Under this limit, δ depends on the size of the relative price shock *S*. When *S *is extremely large (e.g. P_s_(τ) → ∞), then δ → 1. Intuitively, when the spot price is high relative to the target price, even permits associated with full reversals would not be discounted much because the opportunity to shift abatement is valuable when spot prices are considerably higher than expected future prices. This situation is most likely to be encountered during the early periods of a new compliance regime, when technology substitutes, like carbon capture and storage (CCS), may not be widely available. In the opposite extreme, where there is no cost shock whatsoever so that *S *= 1 (i.e. P_s_(τ) → P_e_(τ)), then δ → 0 and no permits would be issued for offsets associated with full reversals, because there is no economic value in moving abatement to future periods when the spot price exactly equals the discounted expected future price.

In applying this rule to cases where P_s_(τ) < P_e_(τ), we recommend that S be set to 1. If the policy allows permits to be banked for future use, as most do, then prices that fall short of expectations probably do not indicate an inefficient time path (such inefficient allocations should largely be arbitraged away through banking), but rather that abatement costs are simply lower than projected. In these circumstances, offset valuations should be based only on the extent to which they result in perfect sequestration, not on broader market conditions.

## Conclusion

The analytic framework described here provides practical guidance to policymakers charged with regulating the future carbon market. Our model is agnostic about the extent to which liability for reversal risk should reside in private or public hands, as long as it is internalized in some way. If internalized privately, then individual entities may use valuation tools like the ones above to determine the optimal use of risky assets within their larger mitigation portfolio. If risk is internalized publicly by discounting *ex ante*, which may be particularly necessary if reversals result from economic leakage off-site rather than physical losses on-site, then the regulator may apply our valuation tools to the project certification process itself. Details of the discounting algorithm could be included in legislation or left to a regulatory body to implement, in either case providing a methodology by which forest carbon and other risky carbon assets could be properly valued and regulated within a future cap-and-trade system.

## Methods

All methods are described in the main text of the manuscript.

## Competing interests

The authors declare that they have no competing interests.

## Authors' contributions

All authors contributed to the intellectual development of the methodology described here. BKM drafted the manuscript and all authors read and approved the final version.
